# Urethral cancer managed with phallus preserving surgery: a case report

**DOI:** 10.1186/s13256-020-02553-z

**Published:** 2021-02-19

**Authors:** Emily Walsh, Niall Kelly, Padraig Daly, Nigam Shah, Ivor Cullen

**Affiliations:** 1 Department of Urology, Department of Surgery, University Hospital Waterford, Waterford, Ireland; 2 Department of Histopathology, University Hospital Waterford, Waterford, Ireland

**Keywords:** Urethral carcinoma, Penis-preserving surgery, Multi-modal therapy, Hypospadic neomeatus

## Abstract

**Background:**

Primary urethral carcinoma (PUC) is rare and accounts for < 1% of all genito-urinary cancers. There is a male predominance of 3:1 and a peak incidence in the 7th and 8th decades. The aetiology of this cancer is similar to penile cancer, and the human papilloma virus (HPV) is thought to be an essential factor in tumorigenesis. Urethral cancer should be diagnosed and staged with a combination of tumour biopsy, MRI, and CT with treatment involving a multimodal approach. Contemporary management emphasises phallus-preserving surgery where feasible.

**Case presentation:**

Here, we describe a case of distal urethral carcinoma, which presented as a metastatic groin mass and identifying the primary lesion proved challenging. Diagnostic flexible cystoscopy identified a tiny lesion in the navicular fossa, which was biopsied and confirmed to be a squamous cell carcinoma. The patient then underwent phallus preserving surgery, including distal urethrectomy with bilateral inguinal lymph node dissections. The final stage was pT1N1M0, and adjuvant chemotherapy was started. The distal urethrectomy involved the surgical creation of a hypospadic meatus in the midshaft of the penis. Normal voiding and sexual function were preserved.

**Conclusions:**

Urethral cancer is a rare malignancy and clinicians should bear in mind that early diagnosis of this disease can be very difficult depending on the anatomical location of the tumour. Treatment currently favours penis-preserving surgery.

## Background

Primary Urethral Carcinoma (PUC) is a rare malignancy, comprising < 1% of all malignancies in male patients. Early reports have identified tumour stage and site as the predominant prognostic factor [1] with higher stage tumours having a worse prognosis as well as those arising in the posterior urethra as compared to the anterior urethra [2]. Urethral carcinoma has historically been managed by partial or radical penectomy for distal tumours or total penectomy with cystoprostatectomy for proximal tumours [3]. However, neoplasms of the anterior urethra can be managed successfully with conservative surgical options [2]. In sharp contrast, the successful management of posterior urethral cancers can prove to be more difficult.

PUCs tend to display a greater variety in histological subtypes, based on the anatomical location and gender. The lining of the urethra alters depending on the anatomical location. This accounts for the variation in tumour subtype. The prostatic urethra is composed of transitional epithelium. In the membranous and spongy urethra there is stratified columnar epithelium. The most common malignant subtypes are squamous cell carcinoma (SCCs) or transitional cell carcinomas (TCCs), with incidences varying in the literature. SCC tends to account for approximately 25% of male urethral cancers and 20% of female urethral cancers [4]. Adenocarcinoma may also occur [5]. SCCs are commonly found in the penile urethra rather than in the bulbar and membranous urethra [6, 7]

The regional spread determines the natural history of urethral cancer at the time of diagnosis. The lymphatic drainage of the anterior urethra is to the superficial and deep inguinal lymph nodes. The main drainage of the posterior urethra is to the pelvic lymph nodes. Approximately one-third of patients may have regional lymph node involvement at the time of presentation [8]. In urethral cancer enlarged lymph nodes are generally indicative of metastatic disease [9]. Although distant metastasis at the time of presentation is rare, this patient presented with a metastatic node left groin. Furthermore, preoperative diagnosis of urethral cancer was difficult in this case due to its non-specific presentation.

## Case presentation

**Fig. 1:**
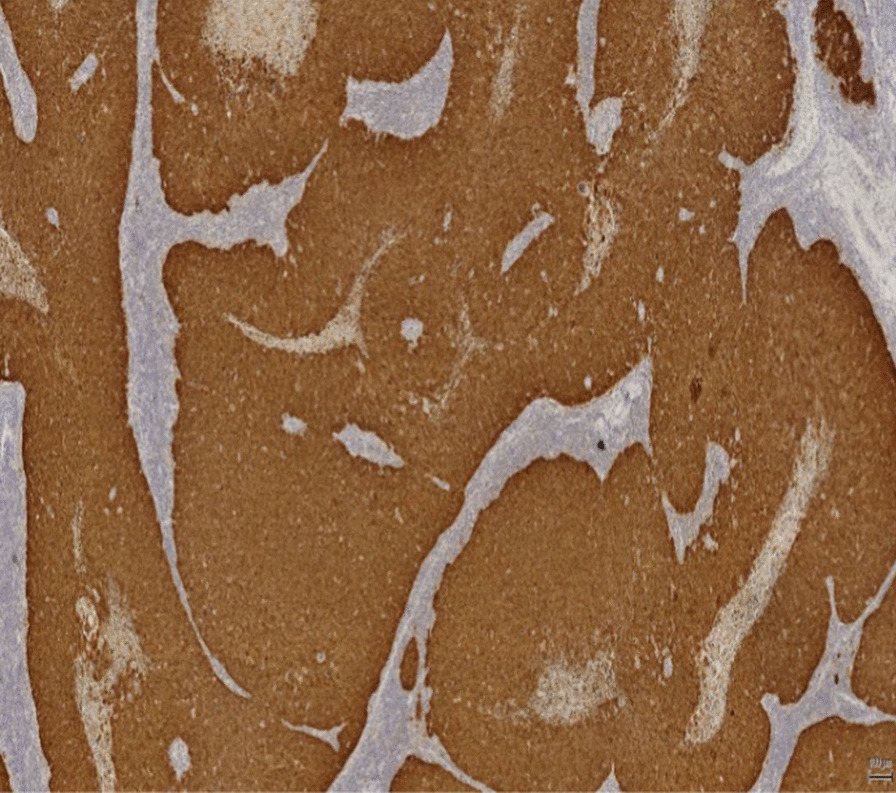
p16 positivity in lymph node metastasis

An 80-year-old Caucasian male initially presented to a peripheral unit with a lymph node mass in left groin. Ultrasound revealed a large pathological lymph node measuring 5 cm in length. A lymph node biopsy was performed and revealed metastatic squamous cell carcinoma with immunohistochemistry findings suspicious for human papillomavirus type 16 specific staining (Fig. 1).

**Fig. 2:**
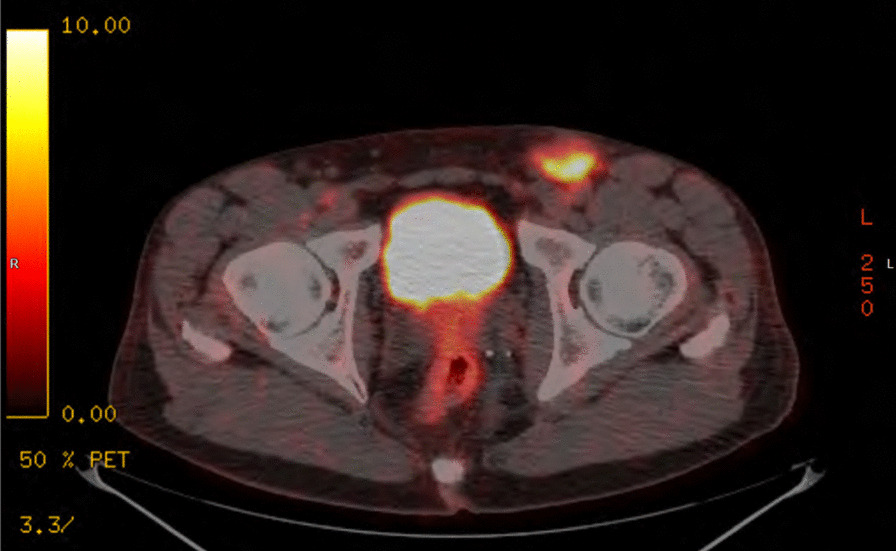
Positron emission tomography scan showing ^18^F-fluorodeoxyglucose uptake in left inguinal lymph node, which was confirmed to have metastatic squamous cell carcinomas

Computed tomographic (CT) scans of the chest, abdomen and pelvis showed bilateral inguinal lymph node more marked on the left and indeterminate subcentimeter left pulmonary nodules. No primary neoplastic process was detected. We performed a 18F-fludeoxyglucose positron emission tomography/computed tomography (18F-FDG PET/CT) which revealed a Standardised Uptake Value (SUV) of 11 on left inguinal mass and low grade uptake overlying lower sacrum (Fig. 2).

**Fig. 3:**
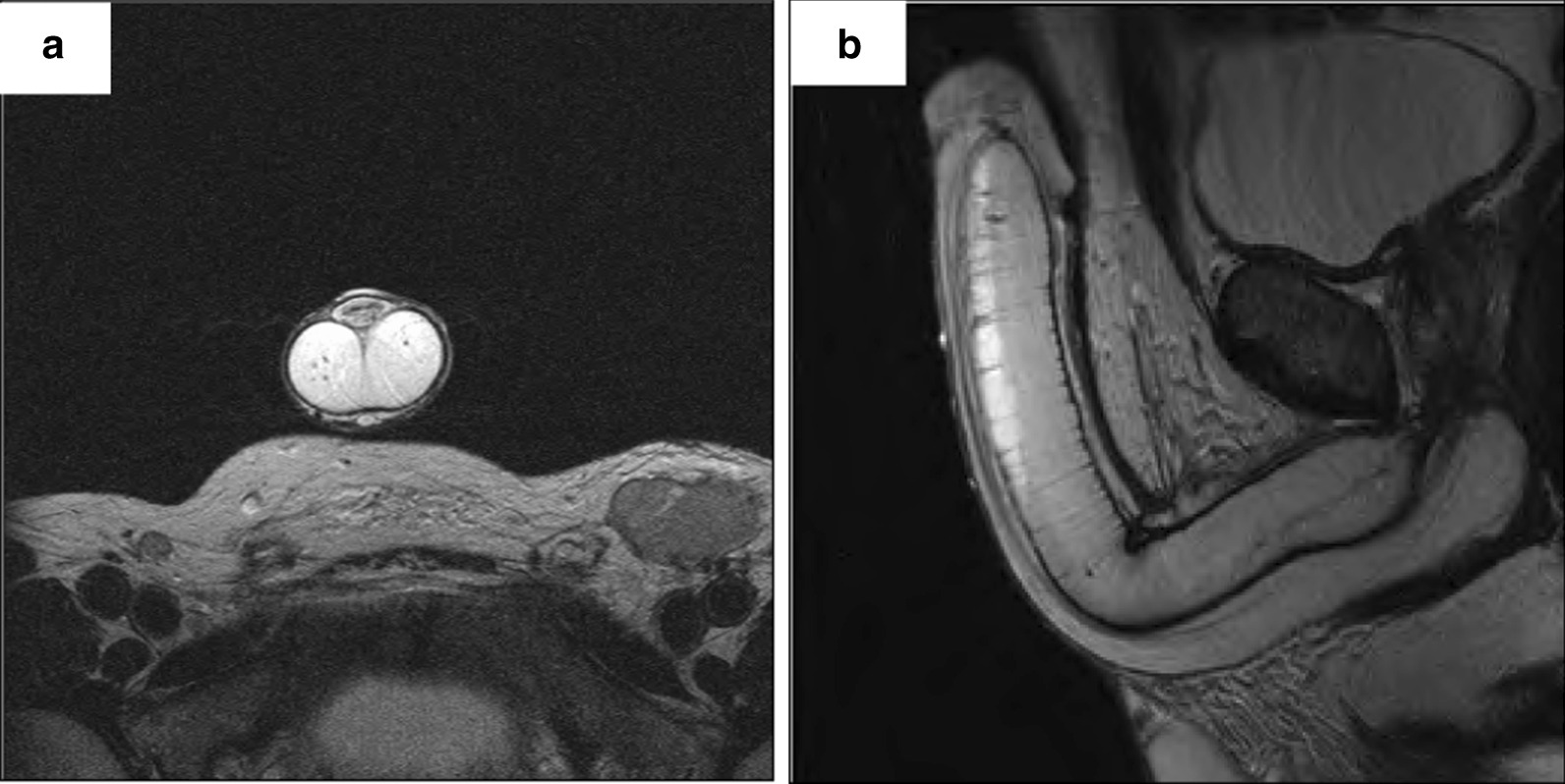
**a** Large lymph node in left groin with normal urethra and corpora cavernosa. **b** Normal corpora cavernosa and normal urethra. Tumour was not evident of the scan

As no primary lesion had been detected and there were histological findings suspicious for a metastatic squamous cell cancer, magnetic resonance imaging (MRI) of the penis was performed to identify a possible penile cancer primary. A T2 weighted MRI (coronal image) after intracavernosal prostaglandin demonstrated a large lymph node in left groin, a normal urethra and corpora cavernosa (see Fig. 3a). MRI after intracavernosal prostaglandin demonstrated normal corpora cavernosa and normal urethra (see Fig. 3b). The use of intracavernosal prostaglandins with penile MRI imaging can help in the detection of penile cancers [8]. No such lesion was identified this patient.

**Fig. 4:**
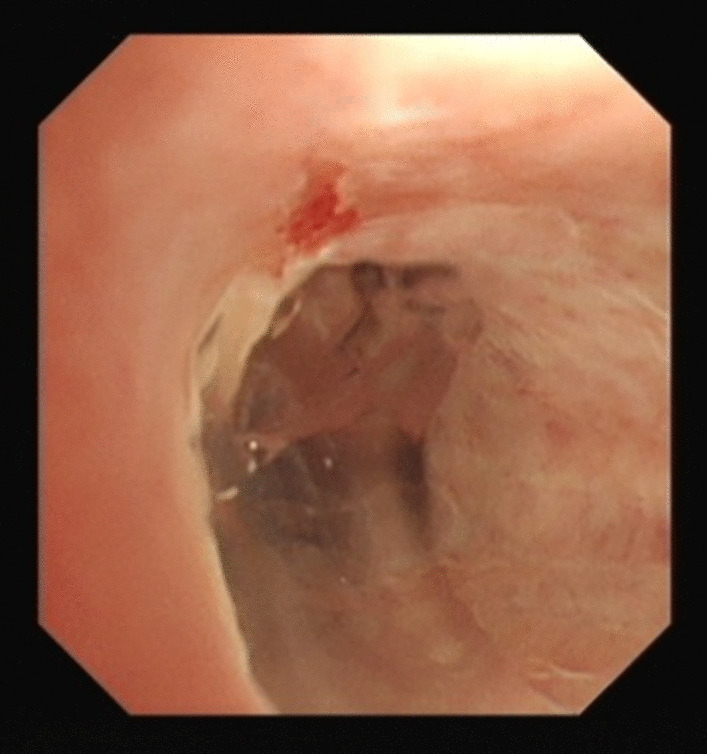
Endoscopic image demonstrating squamous cell carcinomas lesion in the navicular fossa

Proctoscopy and colonoscopy were performed to assess for an anal SCC and were negative for disease. Flexible cystoscopy was also performed which revealed a small nonspecific lesion within the navicular fossa of the distal urethra and was biopsied (see Fig. 4).

Distal urethrectomy with bilateral inguinal lymph node dissection was performed with radical inguinal lymphadenectomy performed on the affected side and a superficial modified approach used on the contralateral groin.

**Fig. 5:**
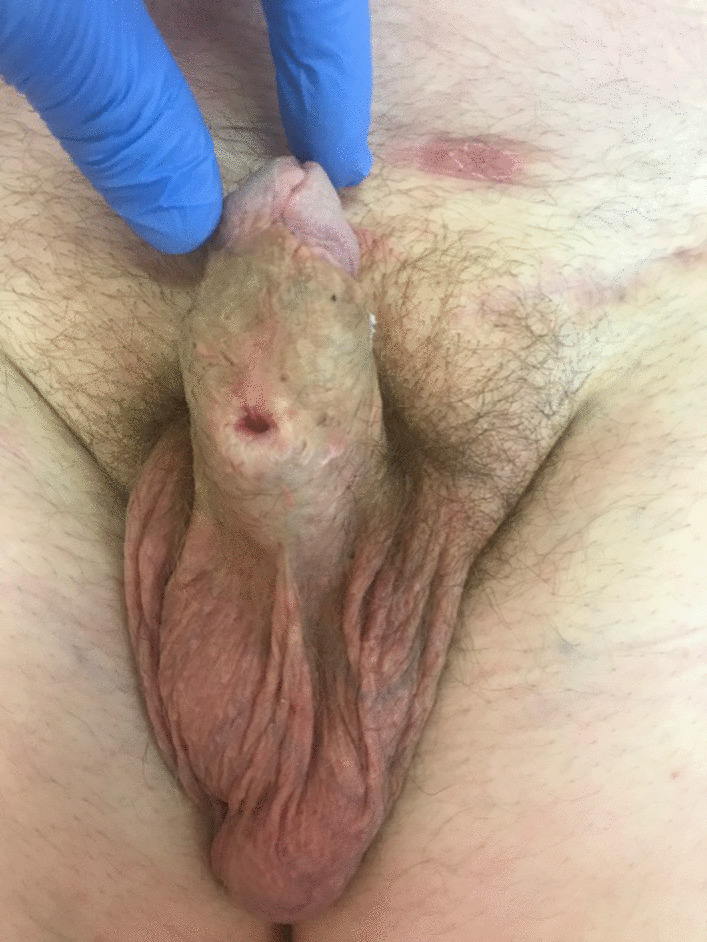
Surgically created hypospadic neomeatus

**Fig. 6:**
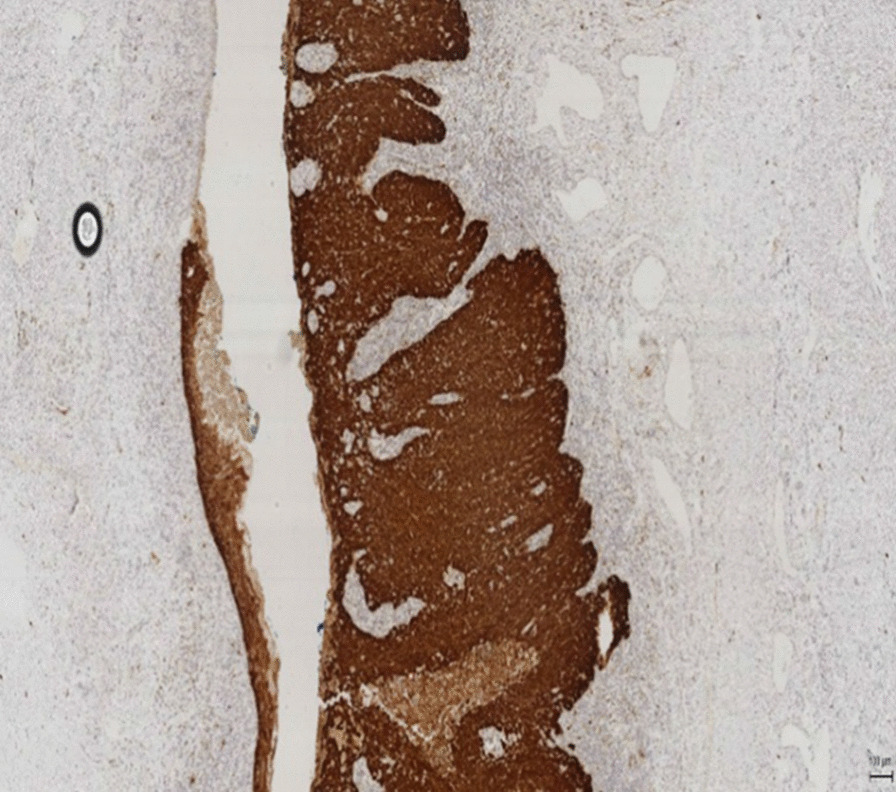
p16 positivity in squamous cell carcinomas

**Fig. 7:**
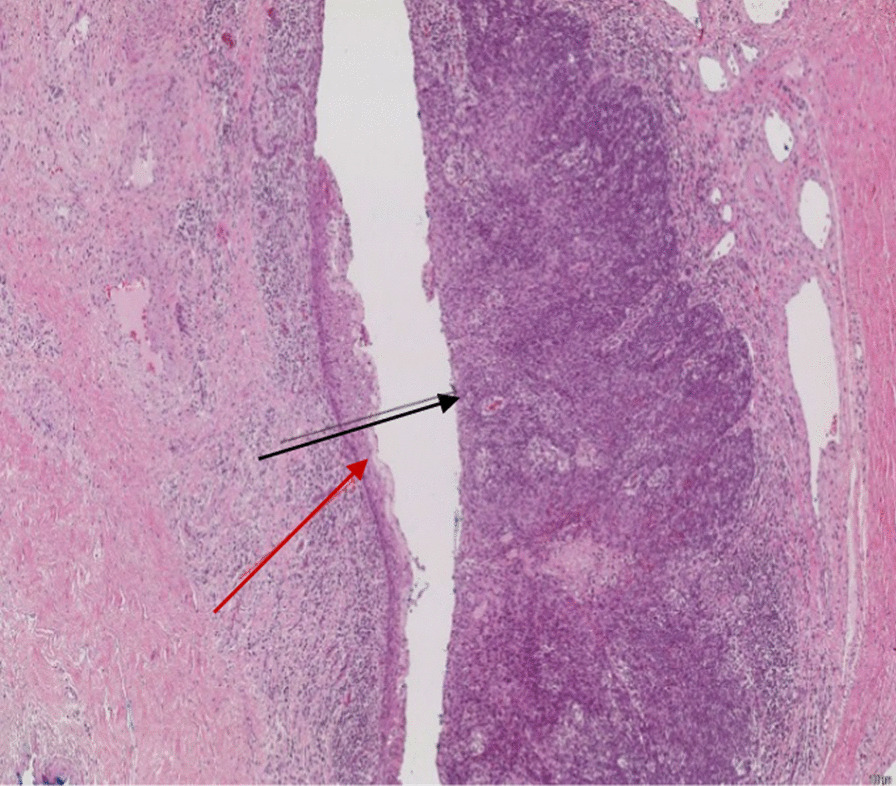
Normal (red arrow) and squamous cell carcinomas (black arrow)

A surgically created hypospadic neomeatus was created within the penile shaft (see Fig. 5). 11 nodes were removed and metastatic squamous cell carcinoma was present in one of the lymph nodes measuring 4.5 cm in length. There was no extracapsular extension. On the right side, 9 lymph nodes were removed with no evidence of neoplasia. Histopathological analysis of the distal urethrectomy specimen confirmed an invasive squamous cell carcinoma of the distal urethra (see Figs. 6 and 7). This was a Human papillomavirus (HPV) related basaloid lesion with an invasive component of 4.5 mm in size. As per the TNM staging, this staged the disease as a T1N1Mx [4]. The patient has been referred for adjuvant treatment consisting of carboplatin (Area Under the Curve (AUC)  = 5) and paclitaxel (175 mg/m^2^) for four cycles.

## Discussion and conclusion

PUC in males often presents with urinary obstructive symptom. One study [10] reported obstructive and irritative symptoms in 43% and 20% of men, respectively. Haematuria, abscess or discharge prior to diagnosis [1] have also been reported as well as a previous history of urethral stricture disease or obstructive symptoms [11–13]. The development of PUC has also been associated with previous urethral surgery and radiotherapy. However, in the case presented here, the patient had no reported symptoms and no risk factors, which made the diagnosis of urethral cancer more difficult. Considering the non-specific presentation of urethral cancer, a high index of suspicion is required for diagnosis.

Due to the low incidence of PUC there is sparse data available on the pathogenesis of urethral cancer. It is believed that chronic inflammation and irritation of the urethra may play a role in the development of urethral cancer. The rapid turnover of the urethral mucosal cells also predisposes to the development of dysplasia and neoplasia. However, the exact pathophysiological mechanisms involved in the development of urethral cancer have not been elucidated.

**Fig. 8:**
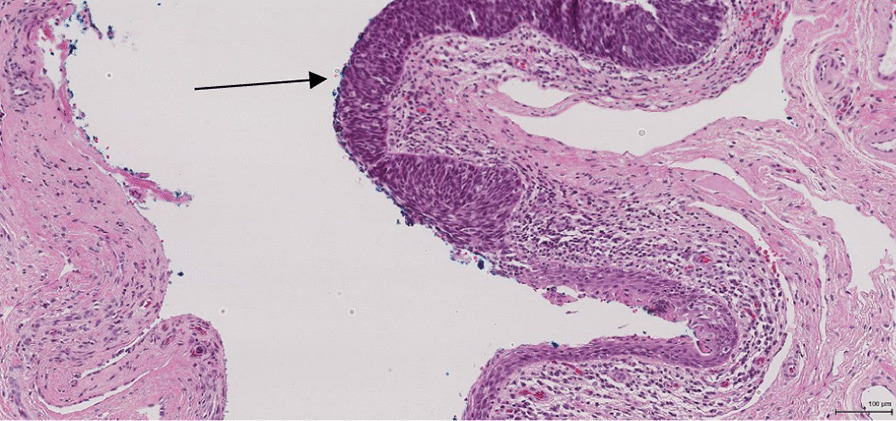
Histopathological picture of urethra with a HPV related basaloid PeIN (arrow) and normal epithelial

**Fig. 9:**
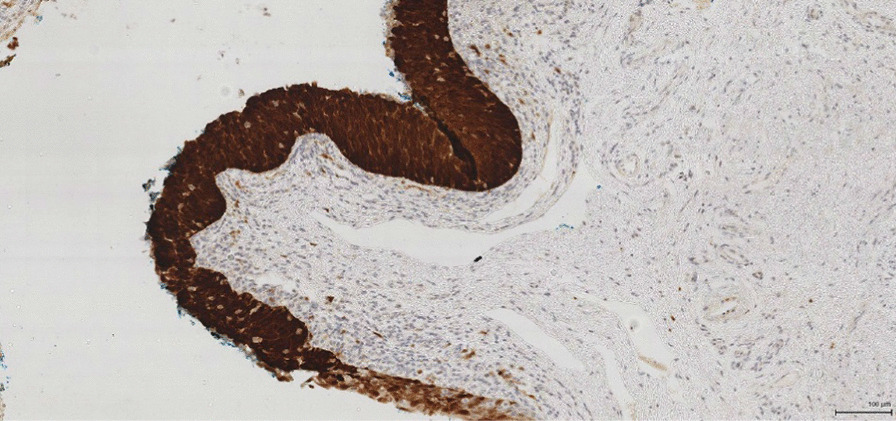
Histopathological picture of penile intraepithelial neoplasia (PeIN) with p16 positivity (brown stain) in the urethral specimen

Approximately a third of urethral cancer cases are due to HPV [14]. The distal urethra has been shown to be a reservoir of HPV infection [15]. The HPV viral proteins E6 and E7 inhibit the actions of p53 and Rb in penile caner [16]. In virally-induced SCC, there is an altered expression of the genes involved in invasion, angiogenesis and metastasis, such as ras, myc and telomerase [17, 18]. In the case presented here, the histopathology report showed an HPV related basaloid lesion in the navicular fossa as well as an SCC in the left groin ( see Figs. 1, 6, 7, 8, and 9). This demonstrates that HPV was the causative agent leading to the development of urethral cancer.

The treatment of urethral carcinoma is not uniform. Two groups of patients with different survival characteristic are recognised. The first group is those with proximal disease. The survival rate is poorer in this group even with a multimodal therapy involving surgery, chemotherapy, and radiotherapy and often presents with advanced disease [6]. The second group is those with distal tumours. These tend to have a better outcome as local surgical control can be achieved [6].

Urethral carcinoma has historically been managed by partial or radical penectomy for distal tumours or total penectomy with cystoprostatectomy for proximal tumours. The management of urethral cancer has been similar to that of penile cancer whereby surgical excision of the primary lesion with a 2 cm clear margin has been encouraged. However, one study showed that < 5 mm resection margins with penis preserving surgery with iliac/inguinal lymphadectomy for clinical suspected nodal disease in men with pT1-3NO-2 anterior urethral cancer resulted in no local recurrence [6]. Thus, phallus preservation surgery can be considered in certain patients. We followed a similar treatment modality for our patient with surgery involving distal urethrectomy with bilateral inguinal lymph node dissections accompanied by chemotherapy. Negative margins were achieved and there has been no local recurrence to date. The outcome was oncologically satisfactory and the patient does not report any complaints about urinary symptoms or erectile dysfunction. Therefore, penis preserving surgeries could be used safely and decrease the psychological burden associated with total or partial penectomy as well as maintaining quality of life and maintaining functional outcome.

Surveillance protocols for patients who have had treatment for urethral cancer has not been established. Tailoring the surveillance regimens based on the patients individual risk factors has been advised [19]. A more extensive follow-up would be required in patients who underwent penis preserving surgery [19]. This includes cytology, flexible cystoscopy and cross sectional imaging. However, evidence that urine cytology may be useful remains to be determined [20]. Our patient’s follow-up will be determined based on response to adjuvant chemotherapy.

We report on the surgical management of a patient with a distal urethral primary carcinoma presenting with local node involvement which was managed successfully with phallus preserving surgery and inguinal node dissection. Primary urethral cancer is a rare urological malignancy that can prove challenging to diagnose and is usually found in the older population. The management of urethral cancer can also be difficult and the more important clinical prognostic factors for male urethral cancer are clinical stage and the anatomical location of the tumour. Treatment currently favours penis-preserving surgery for distal disease, though proximal urethral cancers still require radical resections. The 5 year overall survival is approximately 50%.

## Data Availability

Not applicable.

## Abbreviations

PUC: Primary urethral carcinoma
CT: Computed tomography
HPV: Human papilloma virus
SCCs: Squamous cell carcinomas
TCCs: Transitional cell carcinomas
